# Bacterial Biomarkers of the Oropharyngeal and Oral Cavity during SARS-CoV-2 Infection

**DOI:** 10.3390/microorganisms11112703

**Published:** 2023-11-04

**Authors:** William Bourumeau, Karine Tremblay, Guillaume Jourdan, Catherine Girard, Catherine Laprise

**Affiliations:** 1 Département des Sciences Fondamentales, Université du Québec à Chicoutimi, Saguenay, QC G7H 2B1, Canada; wbourumeau@etu.uqac.ca (W.B.); c9girard@uqac.ca (C.G.); 2 Centre Intersectoriel en Santé Durable, Université du Québec à Chicoutimi, Saguenay, QC G7H 2B1, Canada; guillaume.jourdan@usherbrooke.ca; 3 Pharmacology-Physiology Department, Université de Sherbrooke, Saguenay, QC J1K 2R1, Canada; karine.tremblay@usherbrooke.ca; 4 Research Centre of Centre Intégré Universitaire de Santé et de Services Sociaux du Saguenay–Lac-Saint-Jean (CIUSSS-SLSJ), Saguenay, QC G7H 7K9, Canada

**Keywords:** COVID-19, SARS-CoV-2, oropharynx, oral cavity, microbiome, MiniSeq, biomarkers, dysbiosis

## Abstract

(1) Background: Individuals with COVID-19 display different forms of disease severity and the upper respiratory tract microbiome has been suggested to play a crucial role in the development of its symptoms. (2) Methods: The present study analyzed the microbial profiles of the oral cavity and oropharynx of 182 COVID-19 patients compared to 75 unaffected individuals. The samples were obtained from gargle screening samples. 16S rRNA amplicon sequencing was applied to analyze the samples. (3) Results: The present study shows that SARS-CoV-2 infection induced significant differences in bacterial community assemblages, with *Prevotella* and *Veillonella* as biomarkers for positive-tested people and *Streptococcus* and *Actinomyces* for negative-tested people. It also suggests a state of dysbiosis on the part of the infected individuals due to significant differences in the bacterial community in favor of a microbiome richer in opportunistic pathogens. (4) Conclusions: SARS-CoV-2 infection induces dysbiosis in the upper respiratory tract. The identification of these opportunistic pathogenic biomarkers could be a new screening and prevention tool for people with prior dysbiosis.

## 1. Introduction

In 2020, the World Health Organization declared that the coronavirus disease 2019 (COVID-19), caused by the new severe acute respiratory syndrome coronavirus 2 (SARS-CoV-2) coronavirus, had reached pandemic levels [1]. As of 2023, it has infected more than 761 million individuals worldwide [2]. To date, the scientific community has been working on several fronts to better understand the virus, its health impacts both short- and long-term, and to establish prevention and intervention strategies [3,4,5].

In healthy individuals, the airways harbor a complex community of microorganisms (or microbiome) that contributes to the development of the respiratory tract architecture, plays a role in the development and function of the immune system, and acts as an important component of the epithelial barrier to airway infection [6,7,8]. This has been shown in acute viral respiratory infection, where in this context, the airway microbiome is altered by the viral infection or to support the host immune system response [9,10], which may be promoted by the microbiome via epithelial cells through stimulation of immunocompetence [10,11]. A study on influenza demonstrated that *Corynebacterium pseudodiphtheriticum* in the airways maintains a protective immune environment against viral infections [10]. Conversely, the presence of opportunistic pathogens such as *Streptococcus*, *Haemophilus*, and *Neisseria* can potentially contribute to the development of opportunistic respiratory diseases, thereby leading to more severe respiratory diseases [12,13]. 

A better understanding of the role of the microbiome in relation to the immune response may lead to the use of probiotics to prevent or treat diseases. In this regard, certain taxa in the microbiome could serve as indicators of disease susceptibility and help tailor treatments to restore a more resistant microbial signature to the initial stage of care [14]. Indeed, during viral infections, the oral administration of *Lactobacillus* genera has had a beneficial effect on inducing protective immunity via increased antibody production and natural killer cell (NK) recruitment [15]. Several other examples of the impact of probiotic treatments for respiratory infections or diseases were described in mouse models. Oral administration of *Lactobacillus*, *Bifidobacterium*, and *Lactococcus* strains improves the symptoms of influenza infection by modulating the gut microbiota [16], and the increase in Th2 (responsible for chronic disease) in the blood caused by antibiotics can be prevented by a probiotic treatment [16,17,18]. Those studies showed the impact of probiotics on the gut microbiome but not on the airway one. However, research have demonstrated how the gut microbiome can modulate microbiome diversity in the lung through the gut–lung axis [19,20]. This process includes the oropharynx, which is central to the migration of environmental products and bacteria from one organ to another and illustrates how ingested probiotics can alter the oropharynx microbiome through gut microbiome modifications [21].

In the human airways, the oropharynx harbors the densest and most diverse microbiome [11]. The taxonomic richness of the respiratory tract is at its highest in the oropharynx, primarily because of its unique anatomical location, serving both the digestive and respiratory systems [11]. It is the main source of the pulmonary microbiome in adults, which makes it an interesting region to study as it represents the health status of the airway microbiome [7,11,22]. 

Several studies have been conducted on the impact of microbiome diversity, particularly in the oropharyngeal microbiome, on COVID-19 risk and symptoms. Disruption of the oropharyngeal microbiome in patients with COVID-19 may promote the growth of respiratory pathogens, leading to metabolic imbalances and increasing disease severity [11,23]. Moreover, the composition of the oropharyngeal microbiome is closely linked to the requirement for respiratory support, underscoring the critical role of a healthy oropharyngeal microbiome in the prevention and treatment of COVID-19 [24]. This disruption appears to promote the growth of respiratory pathogens such as *Staphylococcus* and favor the onset of bacteremia following respiratory colonization by *Enterococcus*, suggesting a microbial dysbiosis in patients with COVID-19 [23]. Further studies have demonstrated an inflammatory dysbiosis in patients with COVID-19, marked by the presence of the *Prevotella* and *Veillonella* genera. These findings have been linked to respiratory infections and an increase in the clinical severity of the disease [11,24]. Additionally, the presence of the *Leptotrichia* genus has been associated with the establishment of a pro-inflammatory environment characterized by the production of lipopolysaccharides (LPS) [24]. Furthermore, certain bacteria, such as *Actinomyces*, known for producing mycolic acid, exhibited an inverse correlation with the requirement for respiratory assistance [25]. Gaining a deeper comprehension of the taxa that serve as indicators of COVID-19 risk or severity holds significant importance for this disease, given its high variability in terms of disease presentation and severity of symptoms [11].

In this study, alterations in microbial diversity and the presence of biomarkers in both infected and uninfected individuals were observed, suggesting that viral infection induces changes in the bacterial homeostasis of the respiratory tract. Furthermore, the identification of biomarkers in each group allows us to further our understanding of these links and explore potential prevention strategies for future epidemics. This research is of considerable importance for understanding the interaction between the virus and the microbiome of the oral cavity and oropharynx (OCO), which will enable us to develop targeted measures for preventive public health interventions.

## 2. Materials and Methods 

### 2.1. Study Population

Samples were collected in the Saguenay-Lac-St-Jean (SLSJ) region, in northeastern Quebec (Canada). Population genetics in SLSJ have a unique structure due to several isolated migratory flows [26]. This homogeneity makes it interesting to study genetics, culture, and environmental exposures [27]. This region was largely protected from the initial waves of infection that affected the rest of the province in 2020 due to its remoteness and isolation from large cities, but by the end of 2020, it had infection rates comparable to those of the rest of the province, mainly due to the SARS-CoV-2 alpha variant [28]. 

### 2.2. Samples from the Quebec Biobank of COVID-19

Samples were obtained from screening tests performed by public health officials in SLSJ. From 2020 to 2022, public health guidelines encouraged and/or required the public to get screened to prevent the spread of COVID-19. Individuals were screened following contact with an infected person or if they presented COVID-like symptoms [1]. Screening was performed by a gargle test following the *Centre Intégré Universitaire de Santé et de Service Sociaux* (CIUSSS) protocol and according to the standards [29]. This technique involves gargling the mouth and throat twice with 5 mL of commercial plain water and spitting into a cup, which is then transferred to a plastic tube. These tests were used for PCR screening by Quebec provincial public health to detect the presence of SARS-CoV-2. During these screening tests, each individual had the opportunity to participate in the provincial effort for the study of COVID-19; the *Biobanque québécoise de la COVID-19* (BQC19 which aimed to collect samples and data for research projects [30]. Informed consent for inclusion in the BQC19 was obtained from the individuals being screened or their legal representatives. Ethical approval for the present project was obtained from the Research Ethics Board of the *CIUSSS du SLSJ* (IDs: 2022-388, 2021-026). An aliquot of the gargles was kept for each person tested at the Molecular Biology and Genetics Service, Clinical Department of Laboratory Medicine at the *CIUSSS du SLSJ*, for the purposes of this project. After COVID-19 screening, we had access to 256 samples to conduct the microbiome study. Among them, 182 samples were from individuals who screened positive for SARS-CoV-2, and 74 were from individuals who tested negative. Individuals positive for SARS-CoV-2 were either asymptomatic or presented a mild to moderate form of COVID-19. No individuals included in the study were hospitalized due to COVID-19. The SARS-CoV-2 variant that principally struck Quebec at the time of sample collection was the alpha variant [28].

Participants’ sex and age are presented in Table 1, with age groups classified according to Health Canada’s guidelines [31]. The cohort included 148 women and 108 men, and the average age was similar for men (36 ± 21 years) and women (38 ± 20 years). The positive test (PT) group included 182 individuals (103 women and 79 men) with a mean age of 40 ± 20 years old. There were 74 individuals (45 women, 29 men) in the negative test (NT) group, with a mean age of 30 ± 19 years old (Table 1).

### 2.3. DNA Extraction

DNA was extracted from gargle samples with the DNeasy Powersoil Pro Kit (QIAGEN) following the manufacturer’s recommendations in a Containment Level 2 (CL2) laboratory. DNA was quantified using a fluorometer (Qubit 4) using the Invitrogen™ Qubit™ 1X dsDNA Broad Range (BR) Assay Kit (Life Technologies Corporation, Eugene, United States) [32], with mean DNA concentrations of 6.14 ± 3.14 ng/μL (Supplementary Table S1). Samples were normalized in a 10 mM Tris solution to a concentration of 5 ng/μL for further processing. 

### 2.4. Library Preparation and Sequencing

Sequencing libraries of the V3-V4 regions of the 16S rRNA gene were prepared by 2-step PCR according to the Illumina protocol “16S Metagenomic Sequencing Library Preparation” [33]. Primers used for the first PCR were 341F (5′TCG TCG GCA GCG TCA GAT GTG TAT AAG AGA CAG CCT ACG GGN GGC WGC AG-3′) and 805R (5′-GTC TCG TGG GCT CGG AGA TGT GTA TAA GAG ACA GGA CTA CHV GGG TAT CTA ATC C-3′). The second PCR was prepared with the Nextera XT Index Kit v2 (Illumina, San Diego, CA, USA) [34]. Library size was checked with a Qiaxcel Advanced system (QIAGEN, Hilden, Germany) and confirmed at 550 bp. Libraries were quantified using a fluorometer (Fluoroskan, Thermo Fisher Scientific, Waltham, MA, USA) with the Quant-iT™ 1X dsDNA Assay Kits [35]. Libraries were sequenced on Illumina MiniSeq by paired-end 150 bp cycles. Four sequencing runs were performed on four 96-well plates, generating a total of 47,233,715 reads with a mean of 184,507 ± 3709 per sample. Sequencing details and results are presented in Supplementary Table S2.

### 2.5. Sequence Processing

All analyses were performed with the R environment (version 4.2.0) for sequence processing and all subsequent steps [36]. In order to perform the DNA amplicon sequencing analysis, the DADA2 pipeline was used [37]. Sequence trimming and alignment were performed with the filterAndTrim() function of the dada2 {} package (version 1.24.0). In the trimming step, retained strands were trimmed to a length of 115 bp (truncLen=c(115,115)), no sequences with more than 2 errors were retained (maxEE=c(2,2)), and after trimming, sequences with a quality of less than two were deleted (truncQ=2). This step was performed without removing the primers. Then, errors generated during sequencing were removed (dada()), reverse and forward sequences were merged (mergePairs()), a table with the obtained sequences was created (makeSequenceTable()), sequencing chimeras were eliminated (removeBimeraDenovo()), and taxonomy was assigned to the non-chimeric sequences using a reference database (assignTaxonomy()). All these functions were used from the dada2 {} (version 1.24.0) [38]. Finally, a file to store the DNA sequences thanks to the DNAStringSet()with the Biostrings{} package version (2.64.1) was written [39]. Taxonomic assignment of amplicon sequence variants (ASVs) was performed with the SILVA SSU reference database (version r132_March2018). Finally, the phyloseq() function allowed to create a phyloseq file to facilitate the analysis and the visualization of the data [40]. For all these functions, the parameters have been defined by default. This produced a taxonomic table containing 20,206 ASVs, and samples contained an average of 69,949 ± 28,555 reads. 

The taxonomic table was filtered using the DECONTAM{} package (version 1.16.0) to remove contaminating sequences, which identifies contaminants based on the frequency distribution of each ASV as a function of input DNA concentration [41]. Using the filter_taxa() function, bacterial taxa that have an abundance greater than four counts in at least 10% of the samples are retained; the others are removed [42]. The decontaminated taxonomic table contained an average of 68,284 ± 28,015 reads per sample. These sequences allowed the identification of 9818 ASVs in a total of 256 samples. Multiple rarefaction curves were plotted to ensure that each sample reached saturation of taxa (Supplementary Figure S1). 

### 2.6. Diversity Analyses

The relative distribution of phyla was calculated and compared with a Mann–Whitney test (*p*-value < 0.05). Subsequently, linear regressions were performed on all phyla using the lm() function without multiple correction, to check whether the variables “age” and “sex” and two combined could influence the significant difference in distribution between the PT and NT groups. 

Diversity analyses were also performed in R with the phyloseq{} package (version 1.40.0) [40] and the plot_diversity_stats() function of the microbiomeutilities{} package (version 1.0.16) [43]. Taxonomic richness was calculated with the observed and Shannon diversity indices and compared with a Mann–Whitney test (*p*-value < 0.05) between the PT and NT groups. A Breusch–Pagan test of homoscedasticity (*p*-value < 0.05) was performed using the bp_test() function from the lmtest{} package (version 0.9.40) and on R.

### 2.7. Community Composition across Positive Test (PT) and Negative Test (NT) Groups

Community composition was compared through the Bray–Curtis dissimilarity index and visualized in non-metric multidimensional scaling (NMDS) using the plot_ordination() function of the phyloseq{} package (version 3.3.6). We tested for normality using the Shapiro test with shapiro.test(). Then the distribution of the NMDS data was compared with the Mann–Whitney test with wilcox.test() (*p*-value < 0.05). Analysis of similarity was performed by permutation test with the adonis2() function (*p*-value < 0.05) of the vegan package{} (version 2.6.2) [44]. The measure of the dispersion of the proportions within each sample group was performed by the betadisper() function with the vegan{} package (*p*-value < 0.05; version 2.6.2) [44]. Finally, we carried out permutations with the permutest() function of the vegan{} package (*p*-value < 0.05; version 2.6.2), which allows us to see if there are significantly different distributions between the groups.

### 2.8. Abundance of Specific Taxa in the Positive Test (PT) and Negative Test (NT) Groups

Linear discriminant analysis effect size (LEfSe) was performed using run_lefse(), with the microbiomeMarker{} package (version 1.2.2) [45] to identify the microbial taxa that were features (biomarkers) of the PT and NT groups of participants. The significance threshold for the Kruskal–Wallis test was set with an alpha of 0.05, the significance threshold for the Wilcoxon test was set with an alpha of 0.05, and the LDA log score threshold was four. Taxa scoring higher than four are identified as features.

A heatmap was made to visualize the similarities and differences between the samples according to their taxonomic composition. The prevalence threshold was set to 0.1, which means that only those bacteria that were detected in at least 10% of the samples will be included. It was made using the plot_core() function of the microbiome{} package (version 1.18.0) [46]. A volcano plot was made to visualize differentially abundant taxa across the PT and NT groups, calculated using the ancombc() function of the ANCOMBC{} package (version 1.6.2) [47], with an alpha detection threshold of 0.01 and Bonferroni’s correction for multiple comparisons.

## 3. Results

### 3.1. Firmicutes and Actinobacteriota Dominate the OCO Microbiome

**Figure 1:**
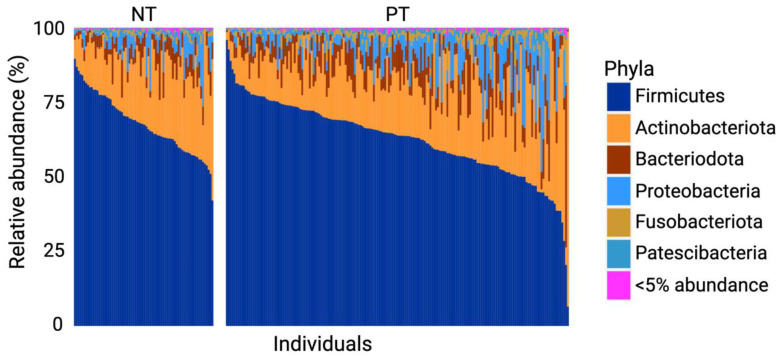
Relative abundance (%) of dominant taxa in samples of the positive test (PT) and negative test (NT) groups. Phyla accounting for less than 5% of all samples were grouped together in the “<5% abundance” group.

For both the PT and NT groups, Firmicutes and Actinobacteriota were the most abundant phylum (with ranges of 56.84–72.48% and 12.82–24.98%, respectively), followed by Bacteroidota (3.47–8.84%), Proteobacteria (1.05–7.54%), Fusobacteriota (0.60–2.02%), and Patescibacteria (0.27–1.16%) (Figure 1).

**Figure 2:**
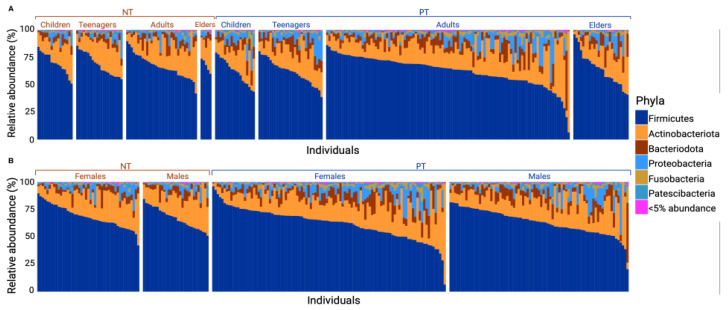
Barplot of relative abundance of phyla in samples of the positive test and negative test groups by sex or age category. This graph shows the relative abundance of phyla present in each sample. (**A**) shows the most abundant phyla among: NT children, NT teenagers, NT adults, NT elders, PT children, PT teenagers, PT adults, PT elders. (**B**) shows the most abundant phyla among: NT Female, NT Males, PT Females, PT Males. Phyla representing less than 5% of all samples were grouped into the “<5% abundance” group.

The relative distribution of phyla varied between the PT and NT groups. Following a Mann–Whitney test, a significant difference was observed in the abundance of Firmicutes (with a mean for PT = 62.68 and NT = 67.83, *p*-value = 0.004), Bacteriodota (with a mean for PT = 7.64 and NT = 5.11, *p*-value = 7 × 10^−4^), and Fusobacteriota (with a mean for PT = 2.04 and NT = 0.95, *p*-value = 7.87 × 10^−8^). Based on linear regression analyses, no significant difference was found in the distribution of phyla abundance between each subcategory within each group (age and sex; see Figure 2A,B).

### 3.2. No Significant Difference in Alpha Diversity between PT and NT Individuals

**Figure 3:**
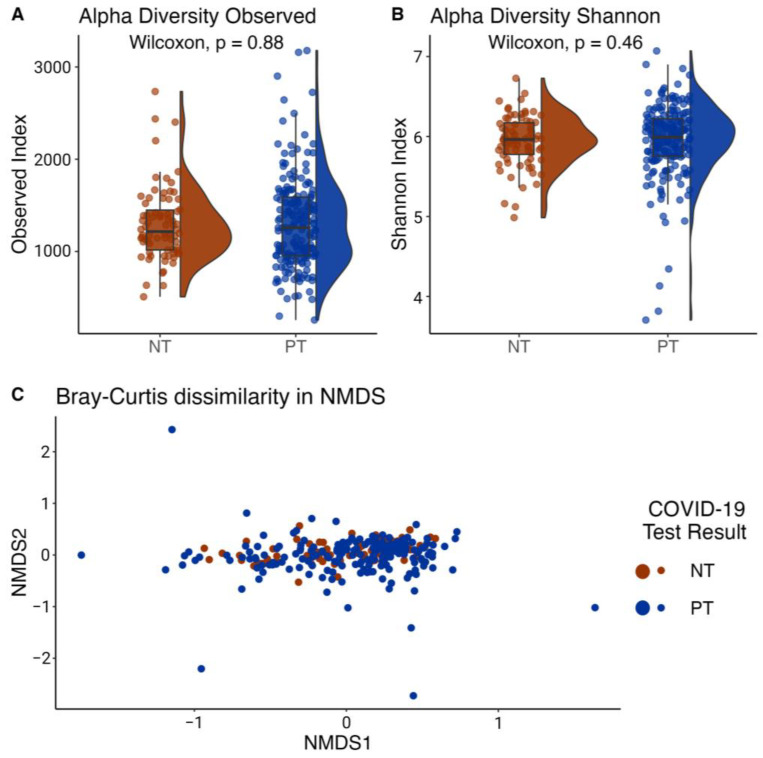
Taxonomic richness and bacterial community composition in samples of the positive test (PT) and negative test (NT) groups. (**A**) Observed and (**B**) Shannon diversity indices. Half-violine plots show the distribution of the calculated index across samples of PT and NT groups. Boxes show quartiles that represent the largest distribution of both sample groups, with the line showing the median. *p*-values reported are from Mann–Whitney tests, with “ns” showing non-significant differences (*p*-value > 0.05). (**C**) Community composition (beta diversity) calculated using Bray–Curtis dissimilarity index in non-metric multidimensional scaling (NMDS) space, for samples of the PT and NT groups.

Alpha diversity indices were employed to compare the taxonomic richness between the PT and NT groups. The results showed no significant difference in taxonomic richness between the two groups (Figure 3A). The mean taxonomic richness based on the observed species index was 1257 ± 510 for the PT group and 1235 ± 400 for the NT group. Similarly, for the Shannon index, the mean was 5.94 ± 0.48 for the PT group and 5.94 ± 0.32 for the NT group. The Wilcoxon test yielded a *p*-value > 0.05 for both the observed index and the Shannon index, indicating no significant difference between the two groups for either index (Figure 3A,B).

### 3.3. Significant Difference in Beta Diversity in Bacterial Communities in Samples of the Positive Test (PT) and Negative Test (NT) Groups

The nonmetric multidimensional scaling (NMDS) graph depicted in our two sample groups (Figure 3C) illustrates the dissimilarity among our samples. After analyzing the bacterial community structure of the OCO, the Bray–Curtis dissimilarity index was calculated. Subsequently, the beta diversity index was employed to compare the taxonomic richness between samples of the PT and NT groups. Given the Shapiro–Wilk test results (*p*-value = 2.749 × 10^−16^), the Mann–Whitney test was used to compare richness between the groups. The results showed a significant difference in taxon richness among our samples between the PT and NT groups (Mann–Whitney test; *p*-value = 0.043).

The adonis2 test shows a variance difference in the taxonomic composition, indicating that the average dissimilarity of bacterial communities was not homogeneous between samples of the PT and NT groups. The screening test variable exhibited an R2 of 2.7% (*p*-value < 0.001). 

Furthermore, the Betadisp index, computed from the Bray–Curtis index, showed a distance of 0.412 and 0.367 between the sample centroids for the PT and NT groups, respectively. The dispersion within each group shows a significant difference (*p*-value = 0.002).

### 3.4. Prevotella and Veillonella Are Features of OCO in COVID-19 Patients

**Figure 4:**
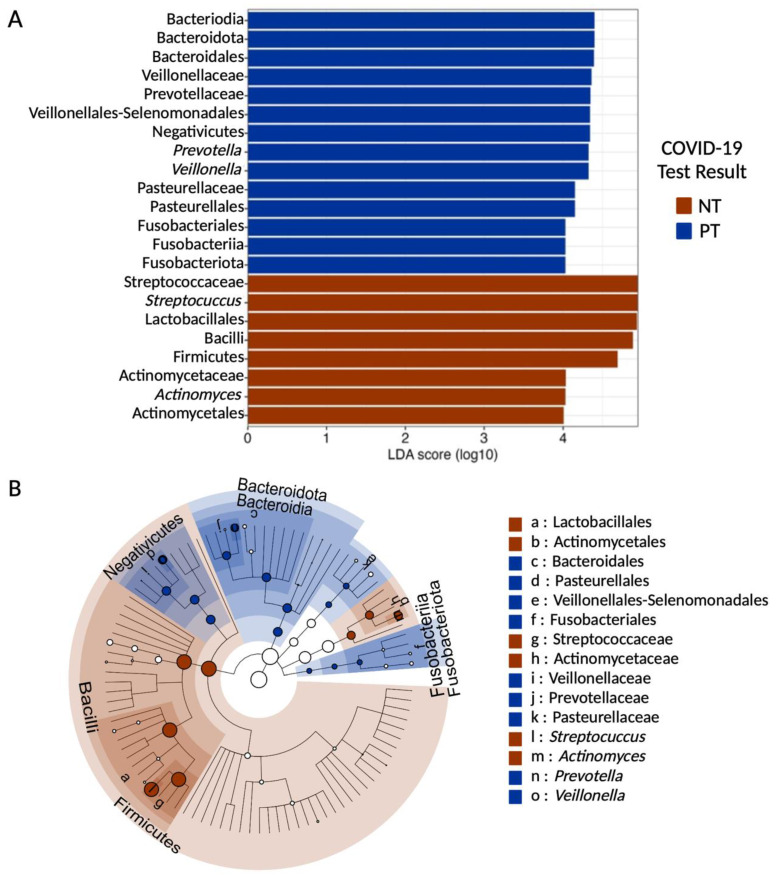
Linear discriminant analysis (LDA) score and cladogram of LEfSe biomarkers. (**A**) Linear discriminant analysis (LDA) effect size (LEfSe) score (minimal score at 4, *p* < 0.05) for taxa identified as biomarkers of either samples of the PT or NT groups. (**B**) Cladogram showing the phylogeny of identified features. Concentric circles represent different taxonomic levels, with the outermost being genus. The nodes (red or blue) represent significantly different features identified as biomarkers. The diameter of each node is proportional to the abundance of the taxonomic rank in the group. Nodes in white are taxonomic ranks that are not significantly different but exhibit differential abundance.

The LEfSe test highlights the bacterial genera most likely to significantly explain the differences between individuals in the PT and NT groups (Figure 4A). 

In this study, the LEfSe test revealed a distinct variation in the relative distribution of genera between our samples from the PT and NT groups. Following a Mann–Whitney test, statistically significant differences were observed for *Prevotella* (mean for PT = 5.54 and NT = 3.27, *p*-value = 1.78 × 10^−5^), *Veillonella* (mean for PT = 6.87 and NT = 4.58, *p*-value = 3.70 × 10^−5^), *Streptococcus* (mean for PT = 49.73 and NT = 58.96, *p*-value = 1.38 × 10^−6^), and *Actinomyces* (mean for PT = 5.08 and NT = 6.16, *p*-value = 8.72 × 10^−3^). These findings indicate that *Prevotella* and *Veillonella* bacteria are features of the PT group, while *Streptococcus* and *Actinomyces* bacteria are characteristic of the NT group.

### 3.5. Individuals with COVID-19 Exhibit Greater Diversity within Their Core OCO Microbiome

**Figure 5:**
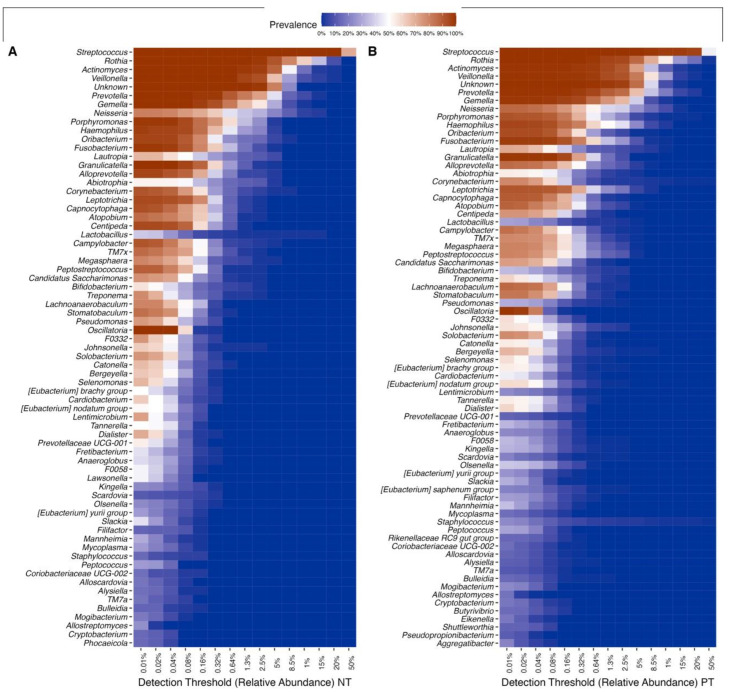
Heatmap of the most abundant genera. Core microbiome genera, shown through their prevalence across samples of the (**A**) NT and (**B**) PT groups (intensity of color) along a step-wise increase in the detection threshold (relative abundance) (*x*-axis).

Of the 106 bacterial genera in the dataset, 38 were identified as part of the core microbiome, common in all samples from both the PT and NT groups. The relative taxonomic diversity between the two communities remained consistent, including Firmicutes, Actinobacteriota, Bacteroidota, Proteobacteria, and Fusobacteriota, which were found in all samples but with variations in prevalence (Figure S2). The most abundant and widespread genera are, in order, *Streptococcus*, *Rothia*, *Veillonella*, *Actinomyces*, *Prevotella*, *Gemella*, *Fusobacterium*, *Granullicatella*, and *Porphyronmonas*, found across all samples (Figure 5). However, the prevalence of these genera differed between the two communities. Overall, samples from the PT group exhibited 74 prevalent taxa, while samples from the NT group showed 69 prevalent taxa, each at a relative abundance of 0.001%.

### 3.6. COVID-19 Infection Status Is Associated with Differentially Abundant Taxa

**Figure 6:**
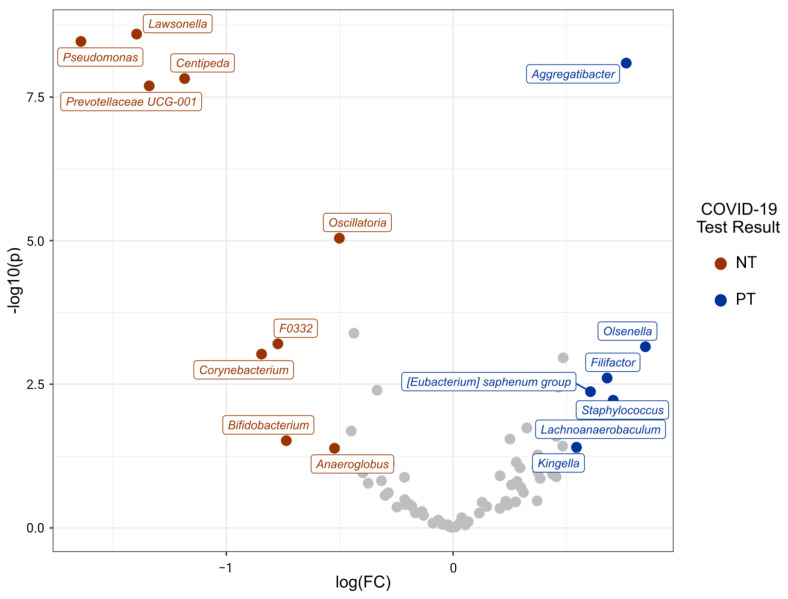
Differentially abundant genera between PT and NT. Volcano plot showing differentially expressed genera between PT and NT groups using the ancombc() function. Genera are colored if they pass the p-log10 value threshold (*p*-value = 0.05) and an absolute log fold change ≥ 0.05. Those that are more abundant in bacterial communities from the PT group are shown in red, while those more abundant in bacterial communities from the NT group are displayed in blue (*p* < 0.01; after Bonferroni correction).

In this dataset, 18 genera were identified as differentially abundant (after Bonferroni correction; *p*-value 0.01; Figure 6). There were 11 differentially abundant genera that were overrepresented in the PT group: *Lentimicobium*, *Lawsonella Pseudomonas*, *Centipeda*, *Prevotellaceae*, *Oscillatoria*, *F0332*, *Corynebacterium*, *Bifidobacterium*, and *Anaeroglobus*. Meanwhile, 7 genera were significantly more abundant in the NT group: *Aggregatibacters*, *Olsenellas*, *Filifactor*, *Staphylococcus*, *Eubacterium saphenum*, *Lachnoanaerobaculum*, and *Kingella*.

## 4. Discussion

The upper respiratory tract is the main entry point for the SARS-CoV-2 virus. After entering the type II epithelial cells, the virus disrupts the homeostasis of the OCO microbiome through viral infection of host cells [48]. This disruption can potentially lead to inflammatory damage, an inadequate immune response, or reduced resilience to the development of COVID-19 [49]. To gain a better understanding of the biology of SARS-CoV-2 infection and its associated symptomatology, this study examined the bacterial communities of the upper respiratory tract and investigated their composition in individuals infected and uninfected with the SARS-CoV-2 alpha variant in the SLSJ region. Early studies conducted at the onset of the COVID-19 pandemic, investigating OCO bacterial communities, did not reveal a significant difference between SARS-CoV-2-infected and uninfected individuals [50]. However, recent studies, including the present one, have demonstrated distinct bacterial communities [23,24,48]. In this study, alterations in microbial diversity and the presence of biomarkers in infected and uninfected individuals were observed, suggesting that the virus induces changes in bacterial homeostasis in the respiratory tract, or that certain microbiome assemblages may promote SARS-CoV-2 infection. While the direction of causality is difficult to identify, this study shows that there are interactions between viral respiratory infections and the OCO microbiome.

A notable strength of this study lies in the fact that the population sampled comes from the SLSJ region, which its geographically remote from the major cities, making it possible to control for variations in the microbiome due to environmental factors (e.g., lower diversity of circulating viruses) or significant cultural differences. Nevertheless, it is important to acknowledge certain limitations associated with the sample composition. Biases in sampling arose because most individuals who underwent COVID-19 testing were either symptomatic, suggesting a proportion of individuals may have tested negative for COVID-19 but may potentially have other unidentified infections. Consequently, interpreting the results was made more challenging by the fact that symptomatic individuals with negative tests might have experienced alterations in microbial diversity due to another respiratory infection. Furthermore, the regular testing of people in the medical field resulted in an overrepresentation of adults and women in the sample. These limitations might also explain why this study did not identify differences regarding age and sex as reported elsewhere [51,52]. It is known that all microbiotas are in development from infancy to adulthood, thus being differently constituted [53]. The underrepresentation of children (0–14 years) in the studied sample might also have contributed to this discrepancy with the literature. Lastly, clinical data such as severity, symptomatology, or outcomes were not available for the studied samples. These data may have been useful for the stratification of the results regarding specific health parameters.

The analyses of phylum abundance revealed that both SARS-CoV-2-infected and uninfected individuals shared the same dominant phyla, with Firmicutes being the most prevalent. Firmicutes encompasses a wide range of taxa in the human commensal flora and has been previously identified as the dominant phylum in both healthy individuals and those infected with COVID-19 in previous studies [7,54,55,56]. The other phyla identified (from most to least abundant: Actinobacteriota, Bacteroidota, Proteobacteria, Fusobacteriota, and Patecibacteria) are also part of the commensal and SARS-CoV-2-infected flora of the OCO [55,56,57]. Among these phyla, a significant decrease in the relative abundance of Firmicutes and a significant increase in Bacteroidota and Fusobacteriota were observed in infected individuals compared to uninfected ones. As these phyla are the most abundant in the OCO, the differences in their relative abundance in infected individuals suggest a state of dysbiosis, indicative of a disturbance in the commensal microbiome. This has been corroborated by other studies that have highlighted an infectious state and a decrease in Firmicutes (see review [58]). Additionally, among the genera belonging to the Firmicutes and Bacteroidota phyla, three were found to exhibit significant differences in their relative abundance and were identified as features of the SARS-CoV-2-infected OCO microbiome. No biomarkers were identified for the phylum Fusobacteriota. However, bacterial genera of this phylum have been shown to produce compounds such as hydrogen sulfide and methyl mercaptan, which, in high concentrations, induce inflammation [59]. Another study shows a negative correlation between the taxon *Fusobacterium periodonticum* and the severity of COVID-19 symptoms [60], indicating the potential importance of this phylum in the immune and inflammatory responses associated with COVID-19 infection.

Despite the decrease in the abundance of the Firmicutes phylum in infected individuals, the genera *Veillonella* and *Prevotella* showed a significant increase in infected individuals and were identified as features for SARS-CoV-2 infection. Both genera are known for their production of LPS [24,25]. LPS, present on the outer membrane of Gram-negative bacteria, can have pro-inflammatory effects on the host immune system and induce systemic inflammation if they are predominant in the microbiome [61,62]. Prior studies have demonstrated correlations between *Veillonella* and *Prevotella* abundances and the severity of COVID-19 symptoms [24,58,63,64,65]. Additionally, individual taxa belonging to these genera, such as *V. parvula*, *V. dispar*, *V. infantium*, *P. enoeca*, and *P. melaninogenica*, were significantly enriched in the OCO microbiome of individuals infected with SARS-CoV-2 and experiencing prolonged symptoms or co-infection with influenza, which can lead to pneumonia [24,66,67,68]. As this study utilized 16S rRNA gene sequencing, which provides information down to the bacterial genus level, specific validation of these results regarding taxonomic abundance in this sample was not possible. However, the strength of this amplicon-based sequencing method is its ability to screen large populations in a cost-effective manner. The results from the present dataset of 256 individuals strengthen observations made on *Prevotella* and *Veillonella*, underscore their contribution in maintaining the commensal OCO microbiome, and suggest they may play a critical role in inflammation in the upper airways. 

The third genus identified as a feature was *Streptococcus* (Firmicutes phylum), which served as a biomarker for uninfected individuals. In this study, a significantly higher proportion of *Streptococcus* was observed in uninfected individuals, contributing to the overall increase observed for this phylum. *Streptococcus* is the most abundant genus in the upper respiratory tract and plays a crucial role in maintaining the homeostasis of the oral microbiome [69]. Microbiomes with a greater abundance of *Streptococcus* and, more specifically, *S. parasanguinis* tended to be more stable and resistant to co-infections or secondary infections, significantly correlating with mild or moderate forms of COVID-19 [66,70]. The depletion of specific *Streptococcus* taxa could indicate a state of dysbiosis of the OCO microbiome, possibly resulting from the overgrowth of other bacteria or an intense immune response [68,71]. This could also potentially be due to direct competitive processes within the OCO microbiome, as observed by the anti-*Streptococcus* effect of host lipids cleaved by *Corynebacterium* [72]. Co-infection with another *Streptococcus* taxa, *S. pneumoniae*, has been found to be one of the most common occurrences following SARS-CoV-2 infection, affecting up to 79% of individuals admitted to the hospital and leading to pneumonia [66,73,74,75,76]. While the functional interactions between *Streptococcus* and SARS-CoV-2 infection could not be assessed from this present amplicon-based analysis, this genus may be an interesting target to develop COVID-19 sensitivity screenings and to better understand the ecological interactions within the OCO microbiome during infection. 

The analyses further identified other possible biomarkers from the aforementioned three phyla. The genus *Actinomyces* was found to be a feature-uninfected individual. While certain *Actinomyces* taxa were associated with mild or moderate forms of COVID-19, a decrease in their abundance was observed in severely affected individuals, and they were also negatively correlated with inflammatory biomarkers such as C-reactive protein [70,77]. On the other hand, the Pasteurellales family and Fusobacteriales order were identified as biomarkers for COVID-19 infection, although no specific genus within these taxa showed significant differences. Nonetheless, genera belonging to these taxa have been found to be significantly increased in cases of co-infection with SARS-CoV-2 [73,78,79,80].

Consistent with previous findings, the results of this study reveal that taxonomic diversity varies in infected individuals compared to uninfected individuals [66,81]. While there was no statistical difference in taxonomic richness between both groups, there were significant differences in community composition, indicating that 2.7% of the variance in taxonomic assemblages of the OCO microbiome was influenced by individuals’ infection status. Given the complexity of the OCO environment and the multitude of variables that could not be included in the analysis (tobacco use, air quality, etc.), this value still represents a significant proportion of the communities. Indeed, in comparison, beta diversity differences in the OCO microbiome for other infectious diseases typically fall below 1% [82,83].

The bacterial communities of infected individuals exhibited greater taxonomic diversity in terms of alpha diversity, and infected samples were more dispersed when visualized in NMDS space. The conventional notion suggests that a healthy microbiome should be characterized by diversity to enhance resilience against disturbances [84,85]. However, stochastic processes that shape bacterial communities can lead to higher diversity than that of a healthy microbiome [66,86]. This observation is further supported by the differential abundance of microbial genera known as opportunistic airway pathogens. Specifically, *Pseudomonas* and *Corynebacterium* were more abundant in the infected individuals, consistent with findings from previous studies [87,88]. These genera are associated with secondary infections in the context of the SARS-CoV-2 infection [87,88,89]. It is essential to note that no causality can be inferred from these observed associations, and they may be influenced by unmeasured confounding factors. 

In conclusion, this study presents the composition and diversity of the OCO microbiome of infected and uninfected individuals with SARS-CoV-2 from the SLSJ region in Canada. The results showed that the OCO microbiome of infected individuals has a different taxonomic composition and diversity, suggesting dysbiosis or increased sensitivity of certain microbiomes to infection. Additionally, four genera have been identified as possible biomarkers for SARS-CoV-2 infection (*Prevotella* and *Veillonella*) and two for the absence of SARS-CoV-2 infection (*Streptococcus* and *Actinomyces*). This study could help to highlight and understand the relationship between the microbiome of the respiratory tract and health in the context of a new pandemic.

## 5. Conclusions

The results of the present study show that the OCO microbiome of infected individuals exhibits a distinct taxonomic composition and community assembly, suggesting dysbiosis. Microbial features such as those identified in this study can be used as biomarkers to implement preventive measures, such as specialized pre/probiotics (for *Streptococcus*), or to identify the most vulnerable individuals in order to apply appropriate treatments (*Veillonella*).

## Figures and Tables

**Table 1:** Age and sex of individuals.

	Positive Test (PT)		Negative Test (NT)		
	182 (71.1%)		74 (28.9%)		
Age group	Men, n (%)	Women, n (%)	Men, n (%)	Women	Total
Children (0–14 years)	5 (2%)	13 (5.1%)	12 (4.7%)	4 (1.6%)	34 (13.3%)
Teenagers (15–24 years)	14 (5.5%)	15 (5.9%)	9 (3.5%)	12 (11.3%)	50 (19.5%)
Adults (25–64 years)	47 (18.4%)	63 (24.6%)	7 (2.7%)	25 (9.8%)	142 (55.5%)
Elderly (65+ years)	13 (5.1%)	12 (4.7%)	1 (0.4%)	4 (1.6%)	30 (11.7%)
Total	79 (30.9%)	103 (40.2%)	29 (11.3%)	45 (17.6%)	256

## Data Availability

We The data presented in this study are available on request from the corresponding author. The data are not publicly available due to due to ethical reasons.

## Supplementary Materials

The following supporting information can be downloaded at: https://www.mdpi.com/article/10.3390/microorganisms11112703/s1, Figure S1. Multiple rarefaction curves. Rarefaction curves showing samples of the positive test (PT) and negative test (NT) groups using the rarecurve() function on R form vegan package{} (version 2.6.2) have been realized from steps of 50. Saturation is close for all our samples, at around 60,000 reads. As a very small minority of samples had less than 60k reads, no rarefaction was applied to retain the maximum amount of information per sample; Figure S2. Heatmap of the most abundant phyla. The heatmap allows us to analyze and visualize the multidimensional data in samples of the negative test (NT) and positive test (PT) groups. The detection threshold of the relative abundance is on the abscissa and the genera on the ordinate. Color shows the prevalence of each phyla in terms of relative abundance. Color intensity shows the percentage in a sample, referring to the prevalence color key; Table S1. Data measured after extraction of gargal DNA, measured at Qubit™ using the Invitrogen™ Qubit™ 1X dsDNA Broad Range (BR) protocol (ThermoFischer Scientific).; Table S2. Sequencing results and number of remaining sequences with each step of data processing from the DADA2 pipeline. (a) denoisedF: number of reads remaining for forward (F) files after the modeling and sequencing error correction (denoising). (b) denoisedR: number of reads remaining for reverse (R) files after the modeling and sequencing error correction (denoising). (c) merged: number of reads remaining after merging the forward and reverse pairs to create a complete sequence. (d) nonchim: final number of reads after elimination of chimeras created during PCR DNA amplification.
