# Supplementary figures and images for: Bacterial Biomarkers of the Oropharyngeal and Oral Cavity during SARS-CoV-2 Infection
Source: Microorganisms. 2023 Nov 4;11(11):2703. doi: 10.3390/microorganisms11112703 (PMC10673573; doi:10.3390/microorganisms11112703)

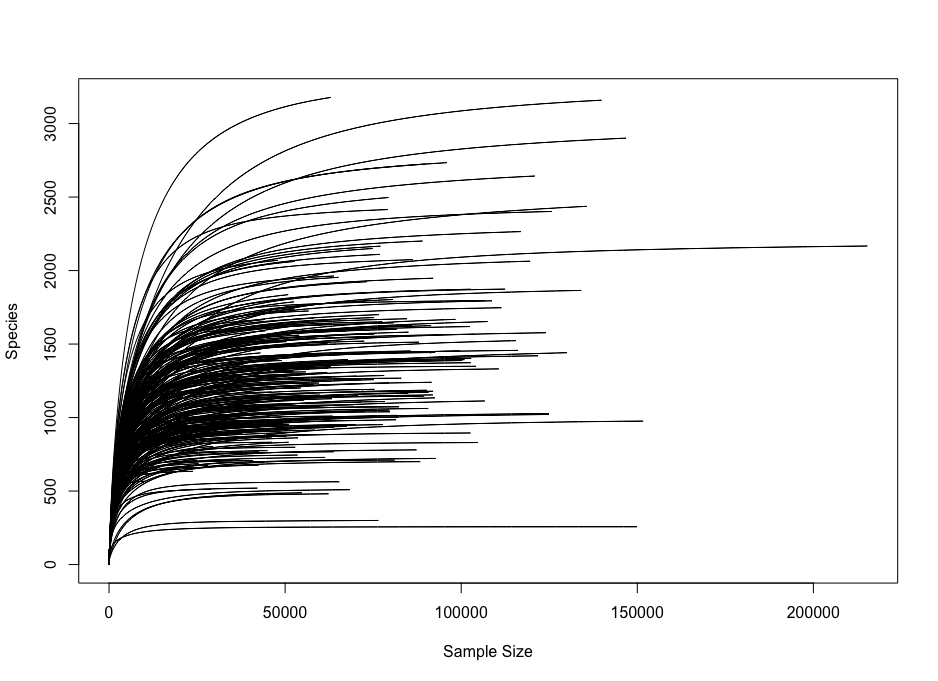

Supplement: Supplementary file 1 [file microorganisms-11-02703-s001.zip › Figure_S1_WB_Rplot.tiff]

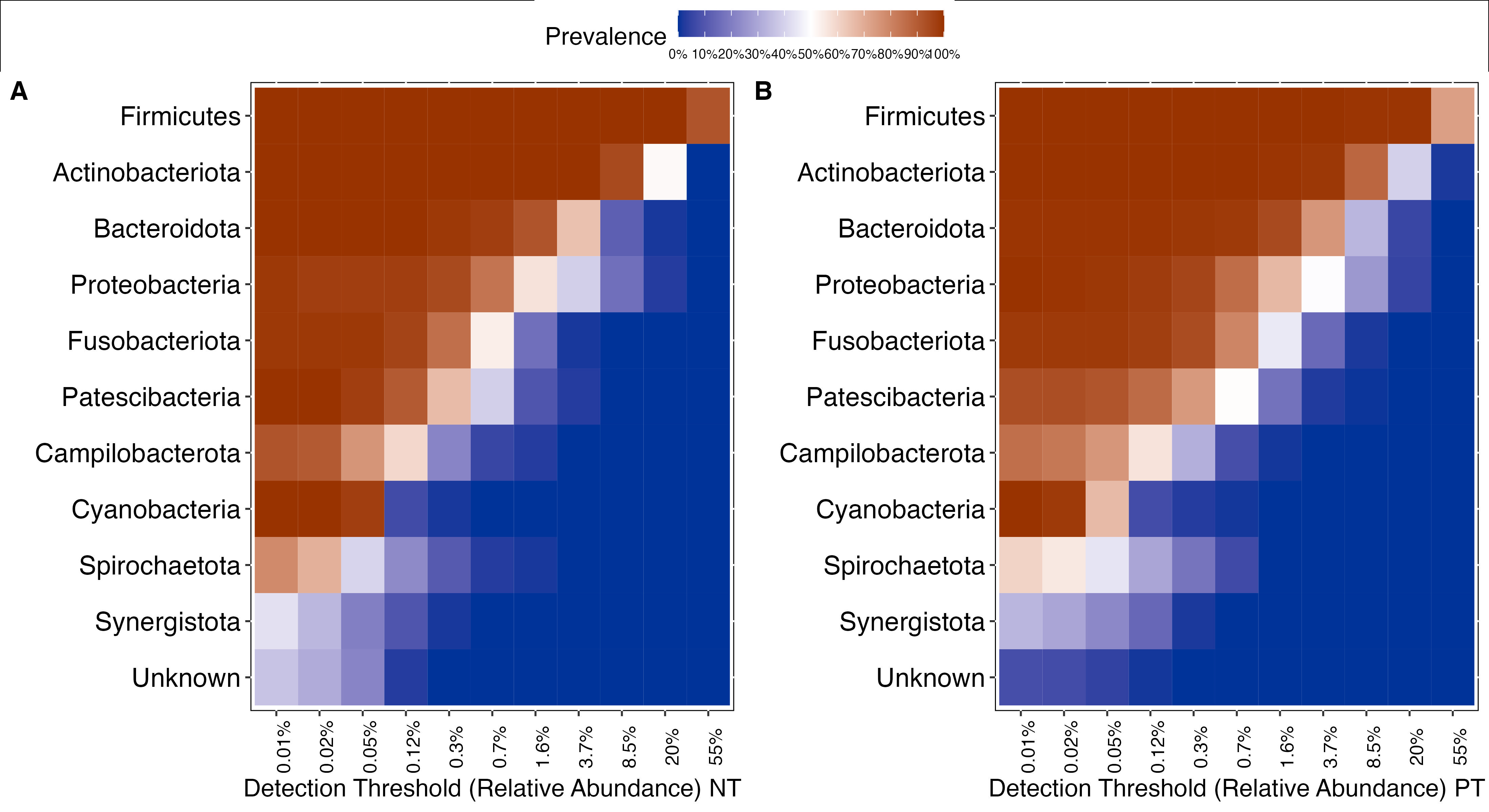

Supplement: Supplementary file 1 [file microorganisms-11-02703-s001.zip › Figure_S2_WB_Heatmap.tiff]
